# A study on the impact of double external shocks on Chinese wholesale pork prices

**DOI:** 10.3389/fvets.2023.1202811

**Published:** 2023-08-21

**Authors:** Rui Yu, Xiaoli Yang, Endong Mu

**Affiliations:** College of Economics and Management, Shenyang Agricultural University, Shenyang, China

**Keywords:** African swine fever, COVID-19, pork prices, wholesale prices, natural experiment

## Abstract

**Introduction:**

Fluctuation in pork prices has always been a focus of academic attention. This paper examines the impact of double external shocks on pork prices, to provide reference for the impact of future outbreaks on the pork market.

**Methods:**

This paper constructs a natural experiment based on the time and regional differences in the occurrence of the epidemics. Double difference models and triple difference models are used to identify the impacts of African swine fever and COVID-19 on Chinese pork prices.

**Results:**

The results found that both African swine fever and COVID-19 positively affected pork prices, but African swine fever had a greater degree of impact; before the COVID-19 epidemic, African swine fever caused a more significant increase in pork prices; the impact of a single African swine fever shock was greater than the double shocks.

**Discussion:**

The COVID-19 epidemic may have curbed the further increase in pork prices, due to the decreased market consumption demand caused by the epidemic.

## 1. Introduction

African swine fever (ASF, the same as below) first emerged in various regions of China in August 2018. Measures, including infection culling, disease-related mortality, and trans-provincial embargoes, severely disrupted the pork market, creating a significant imbalance in supply and demand, and profoundly affected the swine industry (1). The continuous repercussions of ASF were not yet mitigated when COVID-19 (the same as below) emerged in December 2019, exacerbating the impact on pig production, consumption, and pricing (2). As an indicator of economic activity, prices best encapsulate the real-time status of market supply and demand. Agricultural product prices are among the first to be impacted when an epidemic strikes (3). The swine industry and the pork market find themselves in a challenging position, dealing with a complex and overlapping set of external factors. These “double external shocks” have caused instability in the Chinese hog market and further fluctuated pork prices.

Many previous studies have investigated how some epidemic outbreaks can affect the market. Some scholars point out that disease outbreaks can affect hog production (4, 5). Also, epidemics outbreaks can affect the pork trade (6) and lead to bias against the origin (7), while prolonged trade bans can reduce the pork supply (8). After the outbreak of ASF, some scholars have studied that ASF has led to an increase in pork prices and a decrease in demand (9). But currently, research on ASF mainly focuses on the impact of exports, and export losses are the main reason for the increase in the total cost of this epidemic (10). After the COVID-19 outbreak, some scholars pointed out that the COVID-19 epidemic has to some extent disrupted the pork supply chain (5).

Thus it can be seen, the dual external impacts of ASF and COVID-19 are bound to bring a series of serious impacts on the pork market, which will lead to the structural adjustment of the meat market, and the industrial development is full of uncertainty. China is the world’s largest producer of live pigs and consumer of pork (11). The fluctuation of pork prices has always been highly concerned by all sectors of society, ensuring the stability of pork prices, which is related to the well-being of people and the stability of the market. In view of this, the Central Committee of the Communist Party of China and the State Council made an essential deployment in the No.1 central document of the Central Committee in 2020 to ensure the price and stable supply of agricultural products such as pigs, to promote industrial stability and market stability.

Based on previous literature and research findings, many scholars contend that factors influencing pork prices encompass policy regulations, production costs such as feed, transportation expenses, and exogenous shocks (12–15). According to the equilibrium price theory, the key factors affecting pork prices lie in supply and demand. From a supply perspective, production costs and circulation costs can be summarized as internal factors that affect supply, which determine the scale of pig farming. The impact of the epidemic (including disasters) can be summarized as external factors that affect supply, causing great uncertainty to the pig industry and the pork market. There is a wealth of literature on this aspect. For example, Wang Mingli and Xiao Hongbo believe that the impact of the epidemic on the pig market is bidirectional. On the one hand, the epidemic will directly trigger public panic, impact consumer demand, and cause drastic fluctuations in pork prices; On the other hand, the epidemic has led to a decrease in the stock of live pigs, resulting in a decrease in output and impacting market supply (16). Liang Xingqun and Xia Qingli analyzed the chain policy response caused by the epidemic and proposed that the ASF led to market segmentation caused by the government’s implementation of the embargo policy, which restricted the transportation of products between production and sales areas, and subsequently led to drastic fluctuations in pork prices (17).

From the perspective of demand, with the development of the social economy and the increase in people’s income, the demand for pork in the market has undergone profound changes. Drawing on the discussions of Li Binglong and He Qiuhong, from an economic perspective, pork is a normal good with a positive income elasticity of demand. As residents’ incomes increase, so does their demand for pork consumption, leading to higher prices in society. But if, with the development of the economy, residents’ income reaches a higher level, consumers’ consumption habits and concepts are also changing, and the demand for a diversified diet and balanced nutrition increases. Therefore, pork becomes an inferior product, and the effect of income changes on pork prices is exactly opposite to the direction when pork is a normal product. In recent years, the growth in urban residents’ consumption of pork in China has been sluggish, and the high-income group has exhibited a downward trend (18). In other words, an increase in public income is likely to result in a reduction in pork consumption.

In general, there are many studies on the supply and demand situation of the pork market and the impact of the epidemic, but there is still some research space. Previous literature on the impact of external shocks on pork prices has mostly focused on a single external shock perspective, with few discussions on the complex impact of multiple shocks on pork prices. In addition, ASF and COVID-19 in reality occur in a wide range and last for a long time, which provides a place for building Natural experiments, thus providing conditions for analyzing the impact of multiple external shocks on pork prices, which makes the paper supplement and contribute to the previous literature and research theories.

Based on the practical situation described above and the limitations of existing research, we propose the following scientific issues: Since the impact of dual external shocks on the pork market is complex and staggered, to what extent will ASF and COVID-19, respectively, affect the pork market? Is there any difference in ASF impact before and after COVID-19? Will COVID-19 aggravate the impact on the pork market, or will it not cause a new impact? To answer these questions in depth is conducive to sorting out the development and changes of China’s pork market under the dual external impact, and can better explain the phenomenon, reveal the law, and put forward targeted suggestions. This is of great practical significance to ensure the smooth development of the pork market. Considering that price is an important indicator to reflect the stable development of the market, it is feasible and representative to analyze the impact of dual external shocks on the pork market from the perspective of the change in pork wholesale price, and analyzing from this perspective not only has certain practical theoretical significance but also serves as a supplement to relevant literature and theory.

Therefore, under the dual impact of ASF and COVID-19, in the reality of the rapid rise of pork prices, using the data of China’s agricultural wholesale market (sourced from the wholesale market monitoring database of the Ministry of Agriculture and Rural Affairs of the People’s Republic of China), according to the different time differences and regional differences of the two shocks in different regions, Chongqing, Sichuan Province, Zhejiang Province, and Hubei Province were selected to form natural experiments respectively, the difference-in-difference model is constructed to identify the impact of ASF and COVID-19 on the prices of pork wholesale market. On this basis, the triple difference model, replacement sample period, and placebo test are further used for the robustness test. The results showed that ASF and COVID-19 significantly increased the wholesale price of pork; Compared with COVID-19, ASF has a greater impact on the wholesale prices of pork; In addition, the impact of ASF on the wholesale prices of pork is different before and after COVID-19. COVID-19 leads to a decline in market consumption demand, which may inhibit the further increase of pork prices.

Possible contributions of this paper: under the realistic conditions of ASF and COVID-19 double shocks, analyze the changes in pork prices, and analyze the impact of external shocks on pork prices according to the special realistic conditions, which to a certain extent broadens the realistic boundary of factors affecting pork prices; In addition, it is a supplement to the existing literature to study the impact of dual external shocks on pork prices and analyze the impact intensity of different impacts and try to sort out the trend of pork prices changes in complex situations.

The writing structure is as follows: The second part is theoretical analysis; The third part is sample selection, data analysis, and scheme design; The fourth part is the empirical test; The fifth part is conclusions and suggestions.

## 2. Conceptual framework

**Figure 1:**
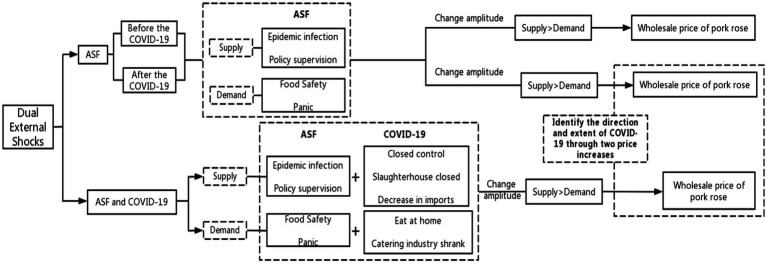
Conceptual framework of the impact of ASF and COVID-19 on the wholesale price of pork.

According to previous studies, the key to the impact mechanism of external shocks on product prices lies in supply and demand. The same is true for ASF and COVID-19. Generally, when a catastrophic or public hazard event occurs, the consumer market is bound to suffer from short-term depression, which will gradually spread to the production end, and then the entire market will experience short-term huge fluctuations. Especially when the external impact directly affects the production end, the whole market will be subject to greater intervention, and the market price change is particularly unstable. To analyze the impact mechanism of ASF and COVID-19 on pork prices, the difficulty lies not only in sorting out the impact of the two on pork prices but also in the complex impact mechanism during the staggered period of the two (see Figure 1).

First of all, the impact mechanism of ASF on pork prices before the occurrence of COVID-19 was analyzed. From the perspective of supply, the sudden outbreak of swine fever has resulted in a significant number of live pigs being infected and many farm households being impacted or even withdrawing from the market, potentially leading to a decline in production (19, 20). At the same time, considering the safety and stability, the government has taken measures such as killing, burning, and burying to further reduce the number of live pigs in the plague area. In addition, pig transportation and other processes are subject to stronger supervision, which increases the difficulty of product supply. The supply of pork in the whole market showed a sharp decline. According to the equilibrium price theory, when supply falls, the supply curve shifts to the left. On the premise of constant demand, prices are bound to rise.

From the perspective of demand, ASF, as an animal disease, greatly affects food safety. Consumers are not aware of swine fever and have consumer panic psychology, which will urge them to change their consumption behavior and seek alternative consumption. In the short term, consumers will increase their demand for alternative products such as poultry and beef, and mutton, thereby driving up the overall price of livestock products (1, 21–23). Although governments at all levels actively publicized ASF-related knowledge, the panic mentality was difficult to eradicate in the short term, the consumer market fell in the short term, and the demand curve also moved to the left. Considering the actual situation, the impact of pig hunting, policy adjustment, and other factors on the pork market is much higher than consumer panic psychological factors, and the degree of left shift of the supply curve may be much greater than the demand curve, so the pork price shows a relatively large upward trend.

Next, the impact of ASF on pork prices during the occurrence of COVID-19 was analyzed. First of all, after the outbreak of COVID-19 (including partial COVID-19 in the post-epidemic era), the local government took closed control measures, blocked transportation roads, and strengthened the supervision of trans-provincial transportation, resulting in traffic disruption. In this case, the transportation of live pigs is blocked, the farms are passively closed, and even some slaughterhouses are closed, affecting the short-term supply of pork. It is worth emphasizing that the supply of pigs is always in a tight state due to the extension of the pig feeding period, and the traffic jam may also further expand the impact of the supply shortage. Due to a series of closed control and transportation blocking measures, at this time, the infection of pig epidemics caused by ASF is reduced, and the government killing is reduced. In addition, considering the poor performance of the international external environment COVID-19, imported pork products are under strict control, further strengthening the uncertainty of pork market supply. That is, from the perspective of supply, under the closed control policy during COVID-19, the impact of ASF was significantly reduced.

In addition, under COVID-19, people are isolated at home, the canteen and restaurants are closed, the people’s food consumption is transferred to their homes, and the catering industry is rapidly declining. Considering the widespread waste of food consumption, the food consumption shift is likely to cause a decline in the market demand for agricultural products. At the same time, the shrinking of the catering industry is bound to have an impact on the agricultural product consumption market. As a major consumer product in the catering industry in China, the demand for pork has declined significantly in the short term. That is, from the perspective of demand, the change in people’s eating habits during COVID-19 has no impact on the pork consumption panic caused by ASF. Therefore, the impact of ASF on the pork demand side has not changed before and after COVID-19.

Finally, analyze the dual impact of ASF and COVID-19 on pork prices. In the COVID-19 state, the market on both sides of supply and demand has shrunk to varying degrees, and the degree of shrinkage is difficult to determine. At this time, there are ASF cases, and measures such as catching and killing are staged again, which will inevitably lead to a stronger impact on the supply side. Therefore, under the double impact of ASF + COVID-19, the degree of impact on pork prices is bound to be higher than that under the single impact of COVID-19. Although Mingli et al. (16) have demonstrated that the impact of epidemic diseases on pork price fluctuations is more severe than that of natural disasters, the severity of COVID-19’s impact has yet to be verified. So, there is uncertainty in the direction of COVID-19 affecting pork prices. The impact of ASF and ASF + COVID-19 on pork prices can be assessed and compared post-COVID-19 to determine the extent and direction of COVID-19’s influence on pork prices. Considering the actual situation, ASF and COVID-19 have a stronger impact on the wholesale price of pork on the supply side. Therefore, the change in supply volume is greater than the demand volume, which ultimately affects the wholesale price of pork and causes price changes. Figure 1 shows the theoretical framework of the impact of ASF and COVID-19 on pork prices.

**Figure 2:**
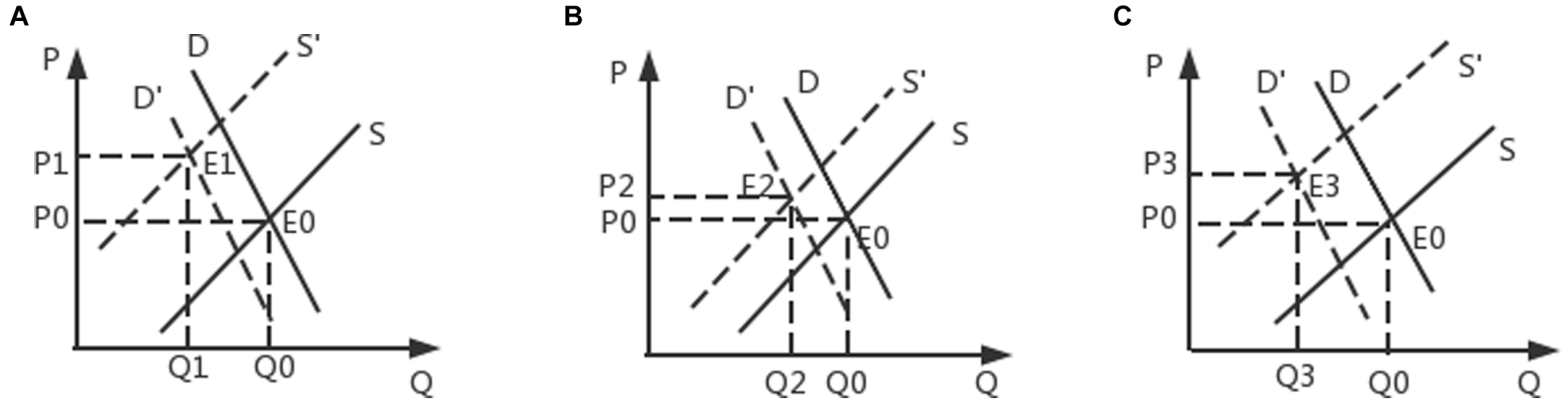
Supply and demand diagram of the impact of external shocks on the wholesale price of pork. **(A)** Effect of ASF before COVID-19 on wholesale price of pork. **(B)** Effect of ASF wholesale price of pork during COVID-19. **(C)** The dual impact of ASF+COVID-19 on the wholesale price of pork.

Next, discuss the relationship between the two from an economic perspective. Draw a diagram of the impact of external shocks on pork prices on supply and demand (see Figure 2). First, analyze the impact of ASF on the pork market (as shown in Figure 2A). Both supply and demand declined. At the supply level, the pork supply decreased sharply, and the supply curve moved to the left, from S to S′. At the demand level, the pork demand decreased due to the substitution transfer of consumption structure, and the demand curve moved from D to D′. Considering that supply is more affected than demand, the supply curve moves farther to the left. Therefore, according to the equilibrium price theory, the equilibrium point E_0_ will move to E_1_, and the equilibrium price will rise significantly from P_0_ to P_1_.

Secondly, analyze the impact of ASF on pork prices during COVID-19 (as shown in Figure 2B). From the supply side, closed control, blocked roads, blocked pig transportation, the passive barrier of farms and the international external environment caused the tight supply of pork market. At this time, the impact of ASF under the background of COVID-19 was weakened by a series of COVID-19 policy measures. At this time, the left shift of the supply curve caused by ASF was smaller than (a); From the demand side, closed control, people are isolated at home, restaurants and canteens are closed, the catering industry shrinks, and the demand for pork market decreases. At this time, the changes in the demand side caused by COVID-19 can not affect people’s panic about the impact of ASF. Therefore, the left shift of the demand curve caused by ASF is the same as that caused by (a). The result shows that in (b), the price of pork rose from P_0_ to P_2_, less than that in (a) (P_2_ < P_1_).

Finally, analyze the role of ASF + COVID-19 dual effects (as shown in Figure 2C). Under the complex influence of COVID-19, curve S is bound to move a large distance to the left by superposing the influence of ASF. At this time, P_3_ is above P_2_ (P_2_ < P_3_), while the influence of COVID-19 on pork price is uncertain, resulting in the single impact of ASF being stronger than the combined effect of the above two types of external impacts (P_3_ < P_1_).

To sum up, in the context of COVID-19, a series of closed prevention and control measures will weaken the impact of ASF from the supply side, so the impact of ASF on the wholesale price of pork before COVID-19 is the strongest, followed by the dual impact of ASF + COVID-19, and finally the impact of ASF on the price of pork during the occurrence of COVID-19, namely P_2_ < P_3_ < P_1_.

## 3. Data and scheme design

### 3.1. Data sources

The data comes from the wholesale market monitoring database of the Ministry of Agriculture and Rural Affairs of the People’s Republic of China, which covers the daily transaction wholesale prices and volume of various agricultural and sideline products. According to the research needs, the daily wholesale prices of pork in all wholesale markets in Chongqing, Zhejiang, Sichuan, and Hubei were selected as the research objects. The data type is daily data, and the period is from March 1, 2017, to March 23, 2020.

In addition, 0–1 dummy variables are used to reflect ASF and COVID-19. When ASF or COVID-19 occurs in the sample period, the indicator is equal to 1, otherwise, it is equal to 0. The deadline for the impact of ASF is based on the release of ASF in the region on the official website of the Ministry of Agriculture; The deadline for COVID-19’s impact is based on the reduction of the risk response level of the notification issued by the region to level 3 or below.

### 3.2. Experimental design

#### 3.2.1. Experimental background

**Figure 3:**
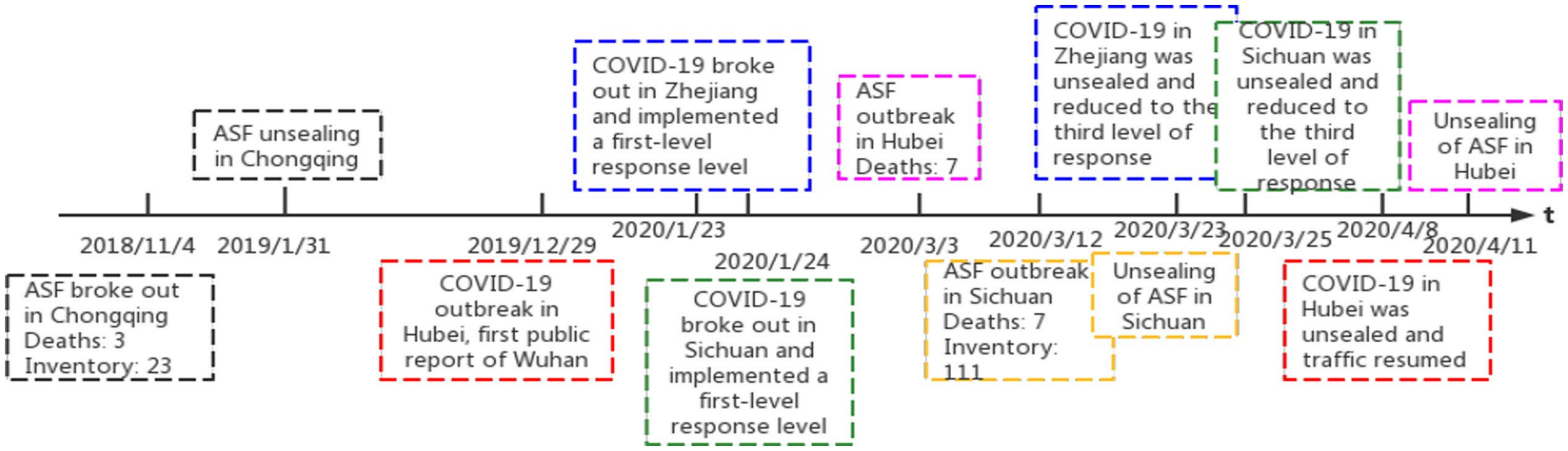
Impact period of ASF and COVID-19 in the sample area. The severity of COVID-19 is divided into four response levels (According to the Emergency Response Law of the People’s Republic of China and the National Emergency Response Plan for Public Emergencies, emergencies are divided into four categories: natural disasters, accident disasters, public health events and social security events. According to the nature, severity, controllability and scope of influence of the event, the emergency level is divided into Level 1 (especially serious) and Level 2 (Major), Level 3 (Major), and Level 4 (General). This COVID-19 is a public health event). The first and second level response refers to the very serious epidemic and the highest risk level; The risk level of Level III and Level IV response is relatively low, and the traffic is restored. Therefore, lowering the response level to Level III and below is COVID-19 unsealing.

ASF and COVID-19 come one after another, affecting most of China. This realistic background provides a natural experimental ground for identifying the impact of dual external shocks on pork prices. First, there are samples of provinces that are impacted and samples of provinces that are not impacted in the same period. The experimental group and the control group can be set up to identify the processing effect of the impact by using the difference-in-difference model; Second, there are differences in the impact time. On the premise that the economic and social development rate of the sample in a single province is consistent year by year, using the method of Hanming (24) for reference, compare the external impact period of a single sample with that of the same period in previous years, and establish an interpreted experimental group and a control group to identify the impact treatment effect (24). Based on this, after sorting out the occurrence of ASF and COVID-19 in each province, three practical situations in line with the research purpose were summarized. See Figure 3 for details.

The first reality is to study the impact of ASF on pork prices before COVID-19. The observation sample area is Chongqing. Since Sichuan Province is a major pork production province (25), Chongqing is located next to Sichuan, which is a typical pork main selling area and has good representativeness. ASF occurred in Chongqing on November 4, 2018, and ended on January 31, 2019. Based on this situation, a single-province inter-temporal difference-in-difference model is constructed.

The second reality is used to study the impact of ASF on pork prices after the COVID-19 epidemic. The observation sample areas are Sichuan Province and Zhejiang Province. COVID-19 of the same level broke out in both places from January 24, 2020, to March 23, 2020. On this premise, the ASF epidemic occurred in Sichuan Province on March 12 of the same year (ended on March 25), but not in Zhejiang Province. Therefore, the daily data were used to construct a control experiment according to the differences between the two places.

The third reality is used to study the dual impact of COVID-19 and ASF on pork prices. The observation sample area is Hubei Province. The province had COVID-19 on December 29, 2019 (unsealed on April 8, 2020) and ASF on March 3, 2020 (ended on April 11), which means that during the period from March 3, 2020, to April 8, 2020, the two impacts caused overlapping impacts. From this, we can also build a single-province inter-temporal difference-in-difference model.

#### 3.2.2. Experimental construction

Based on the above social reality, three groups of control experiments were constructed to identify the impact of external shocks on pork prices in different contexts (see Table 1 for details). The first group of control experiments took Chongqing as the target sample. Referring to the method of Hanming (24), a natural experiment was constructed with the pork price in Chongqing from March 1, 2018, to January 31, 2019, as the experimental group and the pork price in Chongqing from March 1, 2017, to January 31, 2018, as the control group. Among them, during the action period of the experimental group, ASF broke out in Chongqing on November 4, 2018, and ended on January 31, 2019, which can be regarded as the impact action period of ASF, and before the impact action period from March 1 to November 3, 2018. Under the assumption that the social and economic development rate of Chongqing is consistent year by year and the price cyclical change is constrained, the difference between the average price from November 4, 2018, to January 31, 2019, minus the average price from November 4, 2017, to January 31, 2018, and the difference between the average price from March 1 to November 3, 2018, minus the average price from March 1 to November 3, 2017, can be used as a subtraction method to preliminarily identify the effect of ASF on pork price treatment.

**Table 1:** Three groups of control experiments.

Group	Research object	Time
The first group: identified the ASF treatment effect before COVID-19		
Experimental group	Chongqing	March 1, 2018 – November 3, 2018 (before impact)
		November 4, 2018 – January 31, 2019(after impact)
Control group	Chongqing	March 1, 2017 – November 3, 2017 (before impact)
		November 4, 2017 – January 31, 2018 (after impact)
The second group: identified the ASF treatment effect when COVID-19 occurred		
Experimental group	Sichuan	January 24, 2020 – March 11, 2020 (before impact)
		March 12, 2020 – March 23, 2020 (after impact)
Control group	Zhejiang	January 24, 2020 – March 11, 2020 (before impact)
		March 12, 2020 – March 23, 2020 (after impact)
The third group: identified the dual processing effects of COVID-19 and ASF		
Experimental group	Hubei	November 22, 2019 – December 29, 2019 (before impact)
		March 3, 2020 – April 8, 2020 (after impact)
Control group	Hubei	November 22, 2018 – December 29, 2018 (before impact)
		March 3, 2019 – April 8, 2019 (after impact)

The second group of control experiments took Zhejiang Province and Sichuan Province as the target samples. A natural experiment was constructed with the pork price in Sichuan Province from January 24 to March 23, 2020, as the experimental group and the pork price in Zhejiang Province from January 24 to March 23, 2020, as the control group. The above period is in the background of the COVID-19 outbreak (the response levels of the two places are the same). Among them, ASF broke out again in Sichuan Province from March 12 to March 23, 2020, which can be regarded as the impact period of ASF under the background of COVID-19. January 24–March 11, 2020 can be regarded as before the impact period. Assuming that there is a fixed difference in the economy and society between the two places, that is, the difference does not change with time in the short term, and the price fluctuation difference in the short term is constrained, the difference between the average price of pork in Sichuan Province from March 12 to March 23, 2020, and the average price of pork in Zhejiang Province from March 12 to March 23, 2020, is deducted, Subtract the difference between the average price of pork in Sichuan Province from January 24 to March 11, 2020, minus the average price of pork in Zhejiang Province from January 24 to March 11, 2020, to identify the effect of ASF on pork price in the context of COVID-19.

The third group of control experiments took Hubei Province as the target sample. COVID-19 broke out in Hubei Province from January 23 to April 8, 2020, and the ASF epidemic occurred from March 3 to April 8 of the same year. To identify the dual treatment effect of two external shocks, the period of dual impact effect is selected from March 3 to April 8, 2020. Due to the particularity and typicality of the selected samples and the practical constraints, it is difficult to match other sample areas as the control group. We still use the method of Hanming (24) for reference to build a difference-in-difference model based on the sample time difference of a single province. Considering the cleanliness before the policy action period, the pre-action period of the experimental group was from November 22 to December 29, 2019. Similarly, the pork prices of Hubei Province from November 22, 2018, to December 29, 2019, and March 3 to April 8, 2019, were taken as the control group. Under the assumption that the social and economic development rate of Hubei Province is consistent year by year, and the price cyclical change is constrained, the difference between the average price from March 3 to April 8, 2020, minus the average price from March 3 to April 8, 2019, and the difference between the average price from November 22 to December 29, 2019, minus the average price from November 22 to December 29, 2018, can be subtracted to preliminarily identify the treatment effect of ASF and COVID-19 double shocks on pork prices.

## 4. Empirical analysis

### 4.1. Identify treatment effect

According to the above three groups of control experimental design, the difference-in-difference model can be used for estimation. The general model is set as follows.

(1) $$P_{it}=\alpha_{0}+\alpha_{1}time*treat+\theta_{t}+\mu_{i}+\varepsilon_{it}$$

Where, $P_{it}$ refers to the price of pork on the date t of region i. To weaken the possible impact of the pig cycle, the price is logarithmic. *Time* reflects the dummy variable of external impact time, with values of 1 and 0 (1 represents the time of external impact, and 0 represents the time of no external impact). *treat* reflects the dummy variables of the external impact sample, and the values are 1 and 0 (1 represents the area affected by external impact, and 0 represents the area not affected by the external impact). $\theta_{t}$ represents fixed date effect, and we control for temporal factors influencing pork prices, which include macroeconomic shocks, monetary policy, cyclical changes, and prices of substitutes. *μ*_*i*_ represents regional fixed effect, and we control for static regional characteristics such as the level of regional economic development, management expertise, and climatic conditions. $\;\varepsilon_{it}$ is the random error term. The model focuses on the coefficient $\alpha_{1}$ in front of time∗treat , which reflects the effect of external shocks on pork prices.

In addition, the General Office of the Ministry of Ecology and Environment and the General Office of the Ministry of Agriculture and Rural Affairs jointly issued the Notice on Further Standardizing the Delineation and Management of Livestock and Poultry Farming Prohibited Areas to Promote the Development of Pig Production on September 3, 2019, which put forward requirements for pig environmental protection and breeding. This document has brought great intervention in environmental protection, the division into districts, supervision, and other aspects of pig production, and to a certain extent affected the level of pig supply. Therefore, to analyze the relationship between the dual external impact and the wholesale price of pork, we must eliminate the additional intervention brought by the environmental protection policy. In the three groups of control experiments designed, the first group of sample period occurred before the environmental protection policy and was not affected; the second sample period occurs after the policy. Assuming that the control group and the experimental group are affected by the environmental protection policy, the treatment effect in the estimation model (1) is consistent, and the error is negligible; the more complex is the third group of control experiments. The sample period of the experimental group is after the environmental protection policy, while the sample period of the control group is before the policy, which means that the calculated treatment effect may be the result of the triple effects of ASF, COVID-19, and environmental protection policy. Therefore, the difference between the experimental group and the control group in the sample period is controlled, and the impact of the policy is eliminated as far as possible, The effect of introducing the triple difference of annual difference is relatively better.

For the three groups of control experiments, using model (1), the *OLS* estimation without controlling the fixed effect of time and the *FE* estimation with controlling the fixed effect of time were successively used for regression processing. The results are shown in Table 2. Among them, ① and ④ columns reflect the regression results of the first group of control experiments, ② and ⑤ columns reflect the regression results of the second group of control experiments, and ③ and ⑥ columns reflect the regression results of the third group of control experiments. It can be seen from Table 2 that for the first group of control experiments (Chongqing), the effect of ASF on pork price treatment is significantly positive, regardless of *OLS* estimation or *FE* estimation, and through the 1% significance level, it preliminarily shows that the single external impact of ASF significantly promotes the increase of pork price. This conclusion is very close to reality. In particular, as a typical population gathering area, Chongqing has the largest price increase (columns ① and ④ in Table 2 *α_1_* values are significantly higher than other columns).

**Table 2:** Difference-in-difference estimation results.

		OLS		FE		
	①	②	③	④	⑤	⑥
	ASF (Before COVID-19)	ASF (After COVID-19)	ASF and COVID-19	ASF (Before COVID-19)	ASF (After COVID-19)	ASF and COVID-19
Treatment effect	0.2463^***^	0.0478^***^	0.1316^***^	0.2463^***^	0.0300^**^	0.1316^**^
(Treat*Time)	(0.0117)	(0.0109)	(0.0339)	(0.0279)	(0.0132)	(0.0539)
Cons	2.9282***	3.8394***	2.8681***	2.9282***	3.8799***	2.8681***
	(0.0741)	(0.0079)	(0.0114)	(0.1980)	(0.0074)	(0.0140)
*θ_t_*	NO	NO	NO	YES	YES	YES
μ_t_	NO	YES	YES	NO	YES	NO
Obs	674	120	150	674	120	150
R^2^	0.6941	0.7627	0.9873	0.6941	0.6292	0.9873

Robust standard error in brackets.

*** *p* < 0.01.

** *p* < 0.05.

* *p* < 0.1.

The second group of control experiments (Sichuan Province and Zhejiang Province) showed the same status as the first group of control experiments, indicating that ASF in the context of COVID-19 still had a significant positive impact on pork prices. Different from the first group, the columns ② and ⑤ *α_1_* values are far less than that of columns ① and ④. There are two reasons for this situation: first, the regression objects are Sichuan Province and Zhejiang Province (Sichuan Province can be regarded as the main pork-producing province, and Zhejiang Province can be regarded as the main pork-selling province^1^). According to the law of price transmission, ASF leads to a lower rise in the price of pork in the main producing areas than in the main selling areas. Sichuan Province, as an experimental group, was hit by ASF. The impact on its price is relatively small. Second, under the outbreak of COVID-19, social demand fell in the short term, people’s expected income fell, and people tended to hoard durable agricultural products such as grain and edible oil. The closure of restaurants and canteens also led to a decline in demand for meat products. The decline in market consumption demand caused by COVID-19 may be one of the factors that inhibit the further rise of pork prices.

In the third control experiment (Hubei Province), the double impact of COVID-19 and ASF significantly promoted the increase of pork prices. ③ and ⑥ columns *α_1_* values are higher than that of columns ② and ⑤, which shows that COVID-19 is likely to cause the rise in pork prices without considering regional and time differences; However, because of the particularity of Hubei Province in China’s COVID-19 and the fact that its annual pork output is lower than that of Sichuan Province and far higher than the actual structure of the pork market in Zhejiang Province, the strength and direction of the effect of COVID-19 on pork prices cannot be accurately determined. Combined with columns ③ and ⑥ *α_1_* values are lower than the result in columns ① and ④. It can be speculated that after the occurrence of COVID-19, the impact of ASF on pork prices will decrease; However, COVID-19 has an uncertain impact on pork prices, which leads to the result that the single impact of ASF is stronger than the combined impact of the above two types of external shocks.

In general, ASF notably elevates pork prices and exhibits the most substantial effect. However, the impact of COVID-19 on pork prices remains undetermined. This conclusion is consistent with the view put forward by *Xiaohua Yu* in 2020 (26). After the outbreak of COVID-19, the impact of ASF on the wholesale price of pork weakened, and COVID-19 led to a decline in market consumption demand, which may inhibit the further rise of pork prices.

### 4.2. Dynamic effect analysis

The precondition for the coherence of the double-difference estimation results is the fulfillment of the parallel trend hypothesis by both the experimental and control groups (27). This postulates that, in the absence of ASF and COVID-19 impacts, the progression of the outcome variables would have been consistent across both groups. For this reason, this paper empirically tests the dynamic effects of ASF and COVID-19 regarding the *Event Study Approach* proposed by Jacobson et al. (28), and uses the dynamic effect model to estimate. The general model is set as follows:

(2) $$P_{it}=\alpha_{0}+\overset{m}{\underset{1}{\sum }}\alpha_{t}treat*\theta_{t}+\mu_{i}+\varepsilon_{it}$$

**Figure 4:**
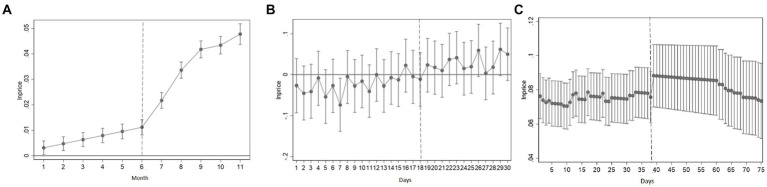
Dynamic effect of double difference. **(A)** ASF (before the COVID-19) dynamic effect. **(B)** ASF (after the COVID-19) dynamic effect. **(C)** ASF and the COVID-19 dynamic effect.

Where, *m* represents the number of days/months in the sample period of the three groups of experimental designs (based on the long sample period of individual natural experiments, it is difficult to present the results in a centralized manner, so the number of months reflects the change). In the first group of control experiments, *m* was 11 in some months of the sample period; In the second group of control experiments, the number of days in the sample period is 30; In the third group of control experiments, the number of days in the sample period is 75. *t* represents time, and the value is between 1 and *m*. In the control experiment of different groups, there are different series of specific estimates, and the definition of other variables is the same as that of the regression model (1). Figure 4 shows the estimated results in three control experiments under a 95% confidence interval.

Figure 4A shows that the change is not obvious in the first 18 months (March 1, 2017, to January 31, 2018), which preliminarily shows that there is no significant difference between the experimental group and the control group before the occurrence of ASF. In addition, the estimated coefficient began to be significant and gradually increased after the occurrence of ASF. Figure 4B shows that before the outbreak of ASF in Sichuan Province on March 12, 2020, there was no significant difference in the wholesale price fluctuation of pork between the two places, and the estimated coefficient was basically below 0, indicating that the pork price in Sichuan Province was lower than that in Zhejiang Province for a long time; After the African pork epidemic, the price difference between the two provinces has changed significantly, with an estimated coefficient of more than 0, and gradually showing a trend that is significantly different from 0. The price of pork in Sichuan Province is significantly higher than that in Zhejiang Province. Figure 4C shows that under the impact of the double shocks, the price of pork rose significantly and remained at a high level for about 22 days, after which the price slowly fell back to the level before the shock.

To summarize, the three control experiments comply with the parallel trend test. Notably, the ASF outbreak preceding COVID-19 had the most significant impact on wholesale pork prices, resulting in short-term continuous increases. The combined impact of post-COVID-19 ASF and the two external shocks induced a relatively moderate effect on wholesale pork prices, which aligns with previous findings.

### 4.3. Triple difference

For the sake of preciseness, considering the possible interference of other unobservable factors (especially the year difference) that affect the wholesale price of pork overtime on the treatment effect, a cross-year triple difference model is constructed to eliminate the above effects. The specific method is to find another pair of “experimental group” and “control group” that is not affected by external shocks in the three control experiments. In the first group of control experiments, another pair of experimental groups and control groups were constructed by pushing forward 1 year. The difference only came from the year and other factors. The difference between the original experimental group and the control group (including the difference of ASF and year and other factors) was subtracted from the difference between the new experimental group and the control group, and the net effect of ASF on pork price was obtained. Similarly, the second group of control experiments (Sichuan Province and Zhejiang Province) and the third group of control experiments (Hubei Province) were both pushed forward by 1 year to construct new experimental and control groups, forming a triple difference, respectively, to obtain the net treatment effect of ASF after COVID-19 and the double impact on the wholesale price of pork. Therefore, the following model is constructed:

(3) $$\begin{array}{l} P_{it}=\alpha_{0}+\alpha_{1}time*treat*group_{1}+\alpha_{2}time*treat\\ \;\;\;\;\;\;\;\;+\;\alpha_{3}time*group_{1}+\alpha_{4}treat*group_{1}+\theta_{t}+\mu_{i}+\varepsilon_{it} \end{array}$$

Where, $group_{1}$ is the newly generated grouping variable. When the newly generated sample data is in the sample period of the original experimental group and the control group, $\;group_{1}$ is 1. When the sample data is in 1 year forward, $group_{1}$ is 0 (the first group of control experimental sample data is from March 1, 2017, to January 31, 2019, $group_{1}$ is 1, and the sample data is from March 1, 2016, to January 31, 2017, $group_{1}$ is 0; The sample data of the second group of the control experiment is 1 in 2020 and 0 in 2019; The data of the third group of control experiment samples is from November 22, 2018, to April 8, 2018, $group_{1}$ is 1, and the sample data is from November 22, 2017, to April 8, 2018, $group_{1}$ is 0). $time*treat*group_{1}$ is 1 indicates the period when the pork price in the region is subject to external shocks. The estimated coefficient $\alpha_{1}$ is a triple difference estimator, representing the average treatment effect of external shocks on the regional pork price. The definition of other variables is the same as that of the regression model (1).

For the three groups of control experiments, using model (3), the OLS estimation without controlling the fixed effect of time and the FE estimation with controlling the fixed effect of time were successively used for regression processing, and the triple difference estimation results were obtained (see Table 3). For the regression results of the first group of control experiments (①④), the coefficient of OLS estimation without controlling the fixed effect of time is 0.2199, which has a significant positive impact. Compared with the coefficient of difference-in-difference estimation, the coefficient of OLS estimation is slightly lower, which is about 2 percentage points lower. However, in the FE estimation with fixed effect of control time, the coefficient of triple difference estimation is slightly higher than the coefficient of difference-in-difference estimation by 0.03; The regression results of the second group of control experiments (②⑤) showed that, like the difference-in-difference results, the treatment effect of external shocks on pork prices was significantly positive, and the degree of treatment effect was lower than that of the first group of control experiments, but the OLS and FE estimation coefficients of the triple difference were slightly higher than the difference-in-difference by 2–3 percentage points; The regression results of the third group of control experiments (③⑥), that is, the treatment effect of double external shocks on pork prices is significantly positive, and the estimated coefficient of the triple difference is about 3% lower than the estimated coefficient of the difference-in-difference, which is slightly larger than the estimated coefficient of the second group of control experiments, and smaller than the estimated coefficient of the first group of control experiments.

**Table 3:** Triple difference estimation results.

		OLS		FE		
	①	②	③	④	⑤	⑥
	ASF (Before COVID-19)	ASF (After COVID-19)	ASF and COVID-19	ASF (Before COVID-19)	ASF(After COVID-19)	ASF and COVID-19)
Treatment effect	0.2199^***^	0.0705^***^	0.0950^***^	0.2771^***^	0.0705^***^	0.0950^***^
(Treat*Time*Group_1_)	(0.0113)	(0.0000)	(0.0077)	(0.0638)	(0.0000)	(0.0118)
Cons	3.1364^***^	2.8994^***^	2.9782^***^	3.1937^***^	3.0118^***^	2.9817^***^
	(0.0044)	(0.0152)	(0.0038)	(0.0641)	(0.0215)	(0.0060)
*θ_t_*	NO	NO	NO	YES	YES	YES
μ_t_	NO	YES	NO	NO	YES	NO
obs	1,011	238	225	1,011	238	225
R^2^	0.6925	0.9408	0.4623	0.7410	0.9174	0.4649

Robust standard error in brackets.

*** *p* < 0.01.

** *p* < 0.05.

* *p* < 0.1.

To sum up, the three groups of control experiments, whether OLS estimation or FE estimation, are consistent with the difference-in-difference results in the direction of action. The effect of external shocks on pork price treatment is significantly positive and passes the 1% significance level. In terms of activity intensity, when excluding the intervention caused by the year difference, the action intensity of ASF and double impact before COVID-19 is lower than that of the difference-in-difference result, the action intensity of ASF after COVID-19 is higher than that of the difference-in-difference result, and the action intensity of ASF after COVID-19 is slightly lower than that of the double impact effect.

The insights derived from this result are: on the one hand, before COVID-19, ASF had the strongest impact on the wholesale price of pork; After COVID-19, the impact of ASF on the wholesale price of pork was significantly reduced (consistent with the difference-in-difference result), and COVID-19 may hinder the further increase of pork price. On the other hand, the impact of dual external shocks on the wholesale price of pork is slightly stronger than that of ASF after the epidemic, indicating that COVID-19 also promotes the increase of the wholesale price of pork, but the impact intensity is slightly smaller. This revelation provides a possible complement to the view of Xiaohua Yu et al. that “COVID-19 has an unknown impact on pork price” (26), and this conclusion is more robust than the double difference result.

### 4.4. Robustness check

#### 4.4.1. Replace sample period

To further prove the reliability and robustness of the results, the method of replacing the sample period is used to test the robustness of the above results. The method is consistent with the construction of the triple difference model. The replacement sample period is to push forward the period of the control group of the three groups of control experiments by 2 years to form a replacement sample, which is tested by the triple difference model. The specific model is consistent with the model (3). The results are shown in Table 4.

**Table 4:** Robustness test.

		OLS		FE		
	①	②	③	④	⑤	⑥
	ASF (Before COVID-19)	ASF (After COVID-19)	ASF and COVID-19	ASF (Before COVID-19)	ASF (After COVID-19)	ASF and COVID-19
Treatment effect	0.2200^***^	0.0705^***^	0.0679^***^	0.2882^***^	0.0705^***^	0.1054^***^
(Treat*Time*Group_1_)	(0.0151)	(0.0349)	(0.0119)	(0.0166)	(0.0346)	(0.0022)
Cons	3.0107^***^	3.0463^***^	3.0451^***^	3.3286^***^	3.0978^***^	3.1205^***^
	(0.0047)	(0.0162)	(0.0042)	(0.0071)	(0.0139)	(0.0044)
*θ_t_*	NO	NO	NO	YES	YES	YES
μ_t_	NO	YES	NO	NO	YES	NO
obs	1,011	238	225	1,011	238	225
R^2^	0.2358	0.9708	0.9812	0.8009	0.9713	0.9904

Robust standard error in brackets.

*** *p* < 0.01.

** *p* < 0.05.

* *p* < 0.1.

For the first group of control experiments (Chongqing), the OLS estimation coefficient without controlling the time-fixed effect is slightly higher than the triple difference by 0.01 percentage points, and the FE estimation coefficient of controlling the time-fixed effect is slightly higher than the triple difference by 0.01, and the results after replacing the sample period are not significantly different; In the second group of control experiments (Zhejiang Province and Sichuan Province), the estimated results after replacing the samples are the same as the above triple difference results, the estimated coefficients are 0.0705, and the estimated results are completely consistent; In the third group of control experiment (Hubei Province), the OLS estimation result is slightly lower than the triple difference by 0.02, and the FE estimation result is only 1 percentage point higher than the triple difference, and the results are almost the same. There is almost no difference between the treatment effect coefficient of the three groups of control experiments and the triple-difference estimation results. The reason is that using the triple-difference model to replace the sample period to eliminate the interference results of uncertain factors such as year can enhance the reliability and explanatory power of the model, but the cross-year, environmental protection policies and the reality of regional development differences in the sample period still affect the results.

The enlightenment is that after the replacement sample period, as with the triple difference result, the action intensity of ASF and double shocks before COVID-19 is lower than that of the difference-in-difference result, and the action intensity of ASF after COVID-19 is higher than that of the difference-in-difference result. After the replacement sample period, the action intensity of a single ASF is still the highest, and COVID-19 has a weak role in promoting the rise of pork prices. The robustness of the above conclusions is confirmed again.

#### 4.4.2. Placebo test

**Figure 5:**
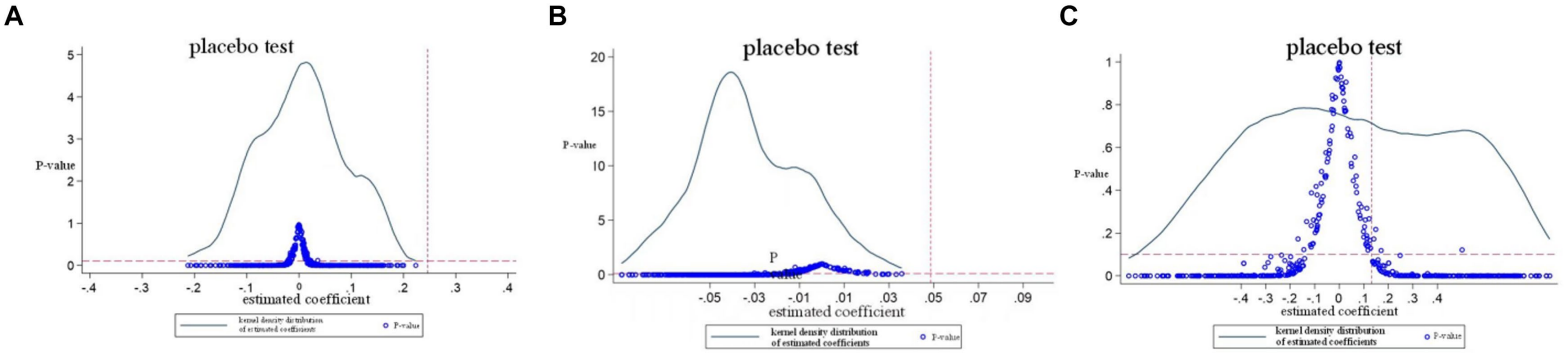
Placebo test. **(A)** ASF (before the COVID-19) estimated coefficients and p-value distribution. **(B)** ASF (after the COVID-19) estimated coefficients and p-value distribution. **(C)** ASF and the COVID-19: estimated coefficients and p-value distribution. The X-axis represents the estimated coefficient of time * treat from 500 randomly assigned. The curve is the estimated kernel density distribution, and the point is the relevant p value. The vertical line is the true estimate of Table 3.

To further test whether the above results are driven by unobservable factors at the year level, a placebo test was conducted by randomly allocating the period affected by external impact factors (29). Specifically, three groups of control experiments were randomly selected as the experimental group in the sample period, and the remaining sample period was used as the control group to construct a double difference model. Random sampling ensures that the constructed independent variable time*treat has no impact on the wholesale price of pork. In other words, any significant findings will indicate that the above regression results are biased. The three groups of control experiments were randomly selected for the length of the sample period with the external impact period (88 days for the first group of control experiment, 12 days for the second group of control experiment, and 36 days for the third group of control experiment), The duration of external impact was randomly selected from three groups of control experimental study sample periods (674 days in total for the first group, 60 days for the second group, and 150 days for the third group) as the experimental group, and the remaining period was the control group. Benchmark regression was performed, the above sampling was repeated 500 times, and the following coefficient distribution and relevant *p* values were drawn (see Figure 5).

The results show that the mean value of the estimated coefficients of all time*treat is almost zero. The distribution of 500 estimated coefficients and their related *p* value distribution are all concentrated near zero, and the p value of most estimated values is greater than 0.1 (not significant at the level of 10%). In addition, the true estimate (from Table 3) is an obvious outlier in the placebo test, which further proves the robustness of the results.

## 5. Conclusions and suggestions

Leveraging data from the wholesale market monitoring database of the Ministry of Agriculture and Rural Affairs of the People’s Republic of China, this paper explores the impact of ASF and COVID-19 on pork prices. By utilizing difference-in-difference and triple difference models, alongside a series of robustness tests, we conduct an empirical analysis of the effects of standalone ASF, and the compound external shocks from ASF and COVID-19 on the wholesale price of pork, leading us to the following conclusions.

First of all, from the direction of action, no matter the single external impact or the ASF and COVID-19 double external impact have a significant positive effect on the pork price treatment, and through the 1% significance level, the external impact promotes the pork price significantly. Secondly, in terms of the impact intensity, before COVID-19, ASF had the highest impact on the wholesale price of pork; After COVID-19, the impact of ASF on the wholesale price of pork decreased significantly, and COVID-19 may hinder the further increase of pork price. Finally, the impact of dual external shocks on the wholesale price of pork is slightly stronger than that of ASF after the epidemic, indicating that COVID-19 also promotes the increase of the wholesale price of pork, but the impact intensity is slightly smaller. The economic implication behind it is that when a catastrophic or public hazard event occurs, the pork consumption market will inevitably suffer from short-term depression, the demand curve will shift to the left, the market will gradually spread to the production end, the supply shortage, the supply curve will shift to the left, and the entire market will experience short-term huge fluctuations. Especially when the external impact directly affects the production end, the whole pork market will be subject to greater intervention, and the market price fluctuation will be greater.

The above conclusions can prove that the external impact is an important factor in the fluctuation of pork wholesale prices, and ASF and COVID-19 have obvious sudden external impacts, resulting in the drastic fluctuation of pork wholesale prices. Therefore, to stabilize the wholesale price of pork and maintain the green and orderly development of the pork market, the following suggestions are put forward based on the current situation.

First, strengthen the early warning mechanism and establish a joint prevention and control system. Improve the prevention and control mechanism of ASF and COVID-19 to prevent the impact of sudden external shocks on the pork market. Improve the emergency response system for major epidemic situations, optimize the internal reporting system of the epidemic situation monitoring and warning mechanism, and ensure that the epidemic situation is grasped at the first time; Improve the joint prevention and control system of the regional epidemic situation and establish the epidemic information sharing mechanism; Build a training system for epidemic prevention and control. Second, we should properly publicize and report, avoid panic, and cultivate market confidence. Strengthen scientific guidance for ASF. Propagandize and guide the ways and means of transmission of ASF, let the public have a scientific understanding, not believe rumors, rationally consume pork and its products, and guide the public to buy pork through formal channels. Third, regulate market prices. External shocks such as ASF and COVID-19 are important influencing factors in pork price fluctuations. Therefore, the government should strengthen the supervision and inspection of the wholesale price of pork and regulate the market price.

## Data availability statement

The data analyzed in this study is subject to the following licenses/restrictions: the price data in this dataset is daily data with a short span. Requests to access these datasets should be directed to XY, yangxiaoli@syau.edu.cn.

## Author contributions

RY and XY: conceptualization, software, and data curation. RY: methodology and writing – original draft preparation. XY: validation. XY and EM: resources and funding acquisition. RY, XY, and EM: writing – review and editing. All authors contributed to the article and approved the submitted version.

## Funding

This research was funded by Research on Countermeasures for the Development of Digital Countryside in Liaoning Province, China (grant number: Z20210226) and Provincial government information system interfacing technical services (grant number: H2021234).

## Conflict of interest

The authors declare that the research was conducted in the absence of any commercial or financial relationships that could be construed as a potential conflict of interest.

## Publisher’s note

All claims expressed in this article are solely those of the authors and do not necessarily represent those of their affiliated organizations, or those of the publisher, the editors and the reviewers. Any product that may be evaluated in this article, or claim that may be made by its manufacturer, is not guaranteed or endorsed by the publisher.

## Footnotes

^1^Sichuan Province, Henan Province, Hunan Province, Shandong Province and Hubei Province are the five provinces with the largest pig production in China. Among them, Sichuan Province has become the province with the largest number of pigs in China with an annual output of 65,791,000 pigs. See link for details https://baijiahao.baidu.com/s?id=1626175637776462025&wfr=spider&for=pc; Tianjin, Jiangsu, Zhejiang, Guangdong and Fujian are the main pork sales areas in the southeast coastal areas of China. See the link for details https://baike.baidu.com/item/ Three-year action plan for accelerating the recovery and development of pig production.
